# Leicester Royal Infirmary

**Published:** 1917-07-28

**Authors:** 


### LEICESTER ROYAL INFIRMARY.

### Work and Progress during 1917.

There were many interesting features in the half-yearly report presented to the governors of the Leicester Royal Infirmary, Mr. T. Fielding Johnson, jun., J.P., presiding. The admissions to the wards for the six months show an increase of exactly 300; the daily average number of beds occupied being 321.8. The pressure on the civil side of the institution has increased, and resort has been had to a waiting list for less urgent cases. It is, however, hoped to overtake the list in the next three months. The out-patients' attendances show an increase of 320, and the accident cases an increase of 823 as compared with the figures of 1916.

### The Hon. Medical Staff.

Owing to military duties, Dr. Bennett, ear and throat surgeon, has arranged with the board to appoint Dr. Macleod to the temporary charge of his department. With the departure of Captain Cumberledge to France, there remain now no assistant physicians or surgeons. Out-patients are for a time to be seen by the senior residents. The resident medical staff and the nursing staff worked loyally for the institution, and earned the gratitude of the governors. Special praise, indeed, is due to the nurses, who were always willing to remain on duty or to get up at any time of the night to receive wounded soldiers on the arrival of convoys.

### Income and Expenditure.

The finances continue satisfactory, but the expenses were exceedingly high. The ordinary income was £17,488 and the expenditure £19,410. Provisions cost

£6,000 for the half-year—an increase of £1,650 on the corresponding period of 1916, drugs and dressings showed an increase of £330, domestic expenses an increase of £750, and salaries and wages an increase of £1,500.

The board has recently had under consideration the payment by the military authorities at the rate of 4s. per day for wounded soldiers, and has unanimously come to the decision to press for a revision of the payment with a view to a considerably increased grant, which they hope will be in harmony with the continuously growing cost of maintenance.

In connection with "Baby Week" the report states that the voluntary hospitals have not received the acknowledgment they deserve for many years' effective work. The Leicester Children's Hospital alone received 120 children of one year and under during the past twelve months. The cost of this treatment was entirely provided out of charitable contributions, not a single penny being directly provided by the Government or by the municipal or county authorities.

Local organisations for the institution throughout the county continue to send generous contributions, and tribute is paid to enthusiastic local organisers, who are real pillars of support of the institution.

SPECIAL DEPARTMENTS.

The pathological department, under Dr. Mackarell, did effective work, and the new department is to be proceeded with directly on the termination of the war. Detail plans and quantities have been prepared by the architect, so that no delay will arise in matters of detail.

The venereal diseases department has become established, and at a recent visit of Sir Arthur Newsholme the county and borough was complimented on being one of the first two provincial centres to become organised. Incidentally, the department has introduced the first lady practitioner to the institution.

FLOURISHING LEICESTER.

The adoption of the report was moved by the Chairman, who pointed out that the figures presented were at once interesting and illuminating, as showing the great part which the voluntary hospitals were continuing to take in the treatment of sickness and disease in its various forms. The cost was heavy, but the trade of the town and county was in a flourishing condition owing to war's demands, and he therefore had no doubt that the necessary income would be provided.

A NEW ELECTRICAL AND MASSAGE DEPARTMENT.

Mr. W. B. Russell (chairman of the house committee) and Dr. Astley Clarke (senior physician) supported the adoption of the report, the latter remarking that he hoped the time was at hand when a new department, specially equipped, would take the place of the present electrical and massage department, which was but an auxiliary to the out-patients' department. He was hopeful that before many weeks had elapsed they would be able to formulate a plan for the establishment of this department.

